# Antibacterial Films Made of Ionic Complexes of Poly(γ-glutamic acid) and Ethyl Lauroyl Arginate

**DOI:** 10.3390/polym10010021

**Published:** 2017-12-24

**Authors:** Ana Gamarra-Montes, Beatriz Missagia, Jordi Morató, Sebastián Muñoz-Guerra

**Affiliations:** 1 Departament d’Enginyeria Química, Universitat Politècnica de Catalunya, ETSEIB, Diagonal 647, 08028 Barcelona, Spain; anagamarramontes@gmail.com; 2 Health and Environmental Microbiology Lab & UNESCO Chair on Sustainability, Universitat Politècnica de Catalunya, ESEIAAT, Ediﬁci Gaia, Pg. Ernest Lluch/Rambla Sant Nebridi, 08222 Terrassa, Spain; beatrizmissagia@gmail.com (B.M.); jordi.morato@upc.edu (J.M.)

**Keywords:** ionic polyglutamic acid complexes, biocide polyglutamic acid, comb-like polyglutamic acid complexes, ethyl lauroyl arginate, antibacterial polymer complexes

## Abstract

The biocide agent LAE (ethyl ^α^*N*-lauroyl l-arginate chloride) was coupled with poly(γ-glutamic acid) (PGGA) to form stable ionic complexes with LAE:PGGA ratios of 1 and 0.5. The nanostructure adopted by these complexes and its response to thermal changes were examined in detail by Differential scanning calorimetry (DSC) and X-ray diffraction (XRD) using synchrotron radiation in real time. A layered biphasic structure with LAE filling the space between the polypeptidic sheets was adopted in these complexes. The complexes were stable up to above 250 °C, non-water soluble, and were able to form consistent transparent films. The release of LAE from the complexes upon incubation in aqueous buffer was examined and found to depend on both pH and complex composition. The antibacterial activity of films made of these complexes against Gram-positive (*L. monocytogenes* and *S. aureus*) and Gram-negative (*E. coli* and *S. enterica*) bacteria was preliminary evaluated and was found to be very high against the formers and only moderate against the later. The bactericide activity displayed by the LAE·PGGA complexes was directly related with the amount of LAE that was released from the film to the environment.

## 1. Introduction

Food safety is today an issue of major concern that is receiving great social and technological attention. It has been estimated that as much as 30% of people in industrialized countries suffer yearly from a food borne disease, and that in 2000, at least two millions of people died from diarrheal diseases worldwide, the major proportion being attributable to microbial contamination of food and water [1]. There are more than 200 of active agents causing gastrointestinal illnesses, about 60% of which being due to infection by food borne bacterial pathogens. *Salmonella* spp., *Campylobacter* spp. and *Escherichia coli* are the bacteria traditionally attracting major attention [2], but in the last decades concerns have included not only an increasing number of additional pathogens as *L. monocytogenes* but also the expansion of modified traditional strains displaying antimicrobial resistance [3]. The use of bactericides, both of synthetic and natural origin, constitutes today the most common practice applied to prevent food spoilage, so the demand for these compounds has increased considerably in these last years [4,5]. Methods followed for impregnating the targeted food with the antimicrobial agent include blending in bulk, surface treatment, and controlled delivery from active films used either for wrapping or coating the food [6,7].

The incorporation of antimicrobials into polymeric films in contact with food to be gradually released during shelf-life has unquestionable advantages over those procedures in which the active compound is directly loaded into or onto the food. (a) Deactivation of the antimicrobial by the food components is largely prevented, and (b) a higher effectiveness in the inhibition of pathogens growing on the food surface, which is the most common way of food contamination, may be achieved. As a result, smaller amounts of active compounds will be required by the film activation approach to reach satisfactory outcomes. This is a very remarkable benefit, since additive minimization constitutes a major challenge today for food quality and safety [8,9].

In this paper we wish to report on a new antibacterial polymeric system based on an ionic polymer complex made of poly(γ-glutamic acid) (PGGA) and a guanidinium-based compound (LAE). PGGA is an emerging biopolymer that is edible and biodegradable, and that has an enormous potential as biomaterial [10]. PGGA is generated by bacterial fermentation of a wide variety of substrates and it is produced at industrial scale to be used as a food complement, in healthcare and for water treatment, among others. As it is much expected for a polycarboxylic compound, PGGA is highly hygroscopic, and a number of modifications, consisting mainly of esterification and amidation of the carboxylic side groups, has been reported with the purpose of making the polymer higher water-resistant [11]. The innocuity of PGGA makes it an excellent candidate for designing antimicrobial polymeric materials for food packaging. On the other hand the polyanionic nature of this biopolymer makes it very suitable for the efficient loading of organocationic compounds by ionic coupling. In fact, ionic complexes of PGGA with both alkylammonium [12,13,14] and alkylphosphonium [15] soaps have been reported, and the capacity of the later to display biocide activity has been demonstrated. Furthermore, the capacity of PGGA to inhibit, by itself, the growth of some pathogenic bacteria has been also announced [16]. Nevertheless the references on the application of PGGA in food packaging are very scarce in the accessible literature [17].

LAE (ethyl ^α^*N*-lauroyl l-arginate) is one of the most potent food preservative agents that is known today, which displays a broad spectrum of activity against food-borne bacteria [18,19]. The high biocide activity of LAE has been attributed to its capacity for altering the metabolic processes of microorganisms without causing cellular lysis [20]. LAE has been assessed to be nontoxic, since after consumption, it is rapidly metabolized to naturally occurring amino acids, among which arginine and ornithine appear to be majority [21,22]. The Food and Drug Administration (FDA) has classified LAE as a GRAS (Generally Recognized as Safe) food preservative at concentrations up to 200 ppm. Antibacterial films containing LAE were firstly prepared from synthetic polymers of common use in packaging such as PP, EVA, and EVOH [19,23,24]. In these last years, efforts has been redirected towards the development of systems made of either bio-based polymers as PLA [25], biopolymers as chitosan [26,27], and others [28,29], which are able to be biodegraded, and even fit to be eaten.

Organocationic compounds are extensively used as bactericides in a diversity of applications, but their utilization in active films is severely limited by the difficulty in achieving suitable mixing with polymers that are commonly used for packaging. Coupling the organocation with anionic polymers is a useful approach that allows for designing active films with the desired stability and releasing properties. The ionic interaction of LAE with anionic polysaccharides has been examined to evaluate the influence that these compounds may have on its biocide activity when they are used as food ingredients [30,31,32]. However, to our knowledge, no study addressed assessing the potential of ionic LAE complexes as active films has been described so far. In this work, LAE has been coupled with the polyanionic PGGA to obtain ionic stable complexes (LAE·PGGA) with antibacterial properties. Firstly, the LAE·PGGA complexes are extensively characterized by physical-chemical methods (Fourier Transform Infrartd (FTIR), Nuclear Magnetic Resonance (NMR), Thermogravimetric analysis (TGA), Differential scanning calorimetry (DSC), X-ray diffraction (XRD), and polarizing optical microscope (POM) to establish their chemical and supramolecular structure. Then, the dissociation of the complexes into their components upon incubation at different pH is examined. Finally the antibacterial properties of the complexes against Gram-positive bacteria (*Listeria monocytogenes* and *Staphylococcus aureus*) and Gram-negative bacteria (*Salmonella enterica* and *Escherichia coli*) are preliminary estimated in order to evaluate their potential for food preserving and packaging applications.

## 2. Experimental Section

### 2.1. Materials

The sodium salt of poly (γ-glutamic acid) (PGGA-Na) sample that was used in this work was kindly supplied by Dr. Kubota of Meiji. Co. (Tokyo, Japan). It was obtained by biosynthesis with a weight-average molecular weight of ~300,000 Da and a d:l enantiomeric ratio of 59:41. Ethyl ^α^*N*-lauroyl l-arginate chloride (LAE) was a sample gifted by Vedeqsa (LAMIRSA Group, Terrassa, Barcelona, Spain).

### 2.2. Measurements

FTIR spectra (Perkin Elmer, Waltham, MA, USA) within the 4000–600 nm range were recorded from powder samples on a Perkin Elmer Frontier equipment provided with an ATR accessory. ^1^H and ^13^C NMR spectra were recorded on a Bruker AMX-300 NMR instrument (Billerica, MA, USA) operating at 300.1 and 75.5 MHz, respectively. Samples were dissolved in deuterated methanol and (tetramethylsilane) TMS was used as internal reference. 128 (Free induction decay) FIDs for ^1^H NMR spectra were recorded with 2.3 μs pulse width, 3.4 s acquisition time, 20 s relaxation delay, and 4.9 KHz spectral width. For ^13^C NMR spectra, 1000 to 10,000 FIDs were recorded using pulse and spectral widths of 4.3 μs and 18 KHz, respectively. TGA was performed at a heating rate of 10 °C·min^−1^ from 30 to 600 °C under an inert atmosphere on a Mettler-Toledo (Zurich, Switzerland) TGA/DSC 1 Star System thermobalance. Sample weights of 10–15 mg were used for this analysis. DSC was carried out on a Perkin-Elmer (Waltham, MA, USA) DSC 8000 instrument that was calibrated with indium and zinc. Heating-cooling cycles at a rate of 10 °C·min^−1^ under a nitrogen atmosphere within the temperature range of –30 to 200 °C were applied for the analysis of sample weights of about 2–5 mg. X-ray diffraction studies were performed using X-ray synchrotron radiation at the BL11 beamline (NCD, Non-Crystalline Diffraction, Cerdanyola del Vallès, España) of ALBA synchrotron in Cerdanyola del Vallès, Barcelona. Simultaneous small angle region (SAXS) and wide-angle region (WAXS) were taken in real time from powder samples subjected to heating-cooling cycles at a rate of 10 °C·min^−1^. The radiation energy employed corresponded to a 0.1 nm wavelength, and spectra were calibrated with silver behenate (AgBh) and Cr_2_O_3_ for small and wide angle diffraction, respectively. Optical microscopy was carried out on an Olympus BX51 POM (Allentown, PA, USA), which was outfitted with a digital camera. For observation, samples were prepared as films casted from 5% (*w*/*w*) methanol solutions and were placed in a Linkam THMS-600 (Tadworth, UK) hot stage provided with a nitrogen gas circulating system.

### 2.3. Complexes Formation and Film Preparation

The methodology originally used by Ponomarenko et al. [33] for the preparation of ionic complexes from poly(α-amino acids) and ionic surfactants were applied in this work. This methodology with some slight modifications has been used previously by us for the synthesis of ionic complexes made from either PGGA [12,13,14] or polyuronic acids [34,35], and quaternary ammonium salts bearing linear alkyl chains with 12–22 carbon atoms. The procedure is essentially as follows: A solution of LAE hydrochloride in water was slowly poured into a solution of PGGA-Na in water under stirring at a temperature around 35 °C. The formed complex precipitated as a white powder after several hours of standing. The precipitate was recovered by centrifugation, and repeatedly washed with water and dried under vacuum for at least 48 h. Complexes were prepared from mixtures containing both 1:1 and 1:2 molar ratios of LAE to PGGA (LAE·PGGA-1 and LAE·PGGA-0.5).

LAE·PGGA films were prepared by casting from a dilute solution of 400 mg of complex in methanol on 3 × 3 cm^2^ Petri plates. After drying at room temperature for 24 h and applying vacuum for 24 h further, consistent films were formed and cut in 1 × 1 cm^2^ squares. The average films thickness measured using a Mitutoyo micrometer (Osaka, Japan) was 100 ± 2 μm.

### 2.4. Complex Dissociation and Antibacterial Activity

The dissociation rate of the LAE·PGGA complexes taking place upon incubation in aqueous medium was followed by measuring the absorbance at 220 nm of the released compounds as a function of time. Assays were carried out by placing 1 × 1 cm^2^ squares of LAE·PGGA films into cellulose dialysis tubes (2000 Da cut-off) that were immersed in 20 mL of buffer solutions at pH = 9.2, 7.4, 5.5, and 4.5 at 25 °C, and were left under mild stirring for one week.

The antibacterial activity of LAE·PGGA complexes was tested in vitro against both Gram-negative and Gram-positive bacteria in liquid culture media over time. Bacteria for this study were selected for their widespread occurrence and well-known ability to cause food-borne diseases by uncontrolled ingestion [2]. Cultures of *E. coli* NCTC 9001 isolated from human urine cystitis, *S. enterica* CECT 4594 from septicemic liver from bovine, *L. monocytogenes* ATCC 19115 from human, and *S. aureus* ATCC 6538 isolated from human lesion were obtained from the National Collection of Type Cultures (NCTC, Public Health England, Porton Down Salisbury, UK), the Spanish Type Culture Collection (CECT, Valencia, Spain), and the American Type Culture Collection (ATCC, Manassas, VA, USA), respectively.

The organisms were stored at −20 °C in tryptic soy broth (TSB; Merck, Darmstadt, Germany) containing 50% (*v*/*v*) glycerol until needed. To activate them, a loopful of each bacterium was streaked on tryptic soy agar (TSA; Difco Laboratories, Livonia, MI, USA) petri dishes. After 24 h at 37 °C, a single colony of each strain was picked and suspended into 10 mL tubes of TSB pH 7 and incubated at 37 °C for 24 h to obtain early stationary phase cells (optical density of 0.9 at 600 nm). The cultures were then further inoculated (100 µL) into fresh TSB and were incubated at 37 °C for 18 h to reach the exponential phase (optical density of 0.2 at 600 nm). At this stage, 100 µL of TSB containing 10^5^ CFU/mL and approximately 1 cm^2^ of each film (PGGA, LAE·PGGA, LAE·PGGA-0.5, and the control) were placed into sterile tubes with 10 mL of fresh TSB and were incubated at 37 °C. For quantification, 100 µL aliquots were removed from the suspension at selected periods of time (2, 8, 24 and 168 h) and plated on petri dishes with 15 mL of TSA culture medium. Serial dilutions were performed with peptone water (1% *v*/*v*) depending on the turbidity produced. Controls without films (blank) and with unmodified PGGA films (negative controls, NC) were also tested and experiments were performed in triplicates. All of the films were sterilized before using by UV light for 15 min. Data are represented as logarithm of colony forming units (LogCFU). Formula for logarithm reduction value (LRV) and percentage reduction calculations are shown below [36],

Log reduction value = log_10_ (*A*/*B*)

Percentage reduction = [(*A*−*B*)/*A*]·100

where *A* is the number of viable bacteria in the negative control and *B* is the number of viable bacteria after treatment with either LAE·PGGA-1 or LAE·PGGA-0.5.

## 3. Results and Discussion

### 3.1. Synthesis of Complexes

**Scheme 1:**
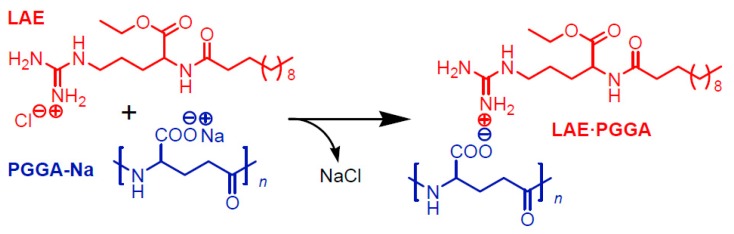
Coupling reaction leading to ionic ionic stable complexes ((ethyl ^α^*N*-lauroyl l-arginate chloride) LAE·PGGA (poly(γ-glutamic acid)) complexes.

The synthesis of the complexes of PGGA and LAE did not entail any special difficulty since they were spontaneously formed upon mixing the aqueous solutions of LAE and PGGA-Na (Scheme 1). Ionic coupling of the guanidinium cation of LAE and the carboxylate anion of PGGA resulted in non-water soluble stable complexes that precipitated from the aqueous solution upon standing. Two LAE:PGGA ratios, i.e., 1:1 and 1:2, were used with the purpose of evaluating the effect of composition on properties. The complexes were recovered by centrifugation in the form of white powders in 50–70% yields. Conditions that were used in these experiments and results attained are given in Table 1.

### 3.2. Chemical Characterization

**Figure 1:**
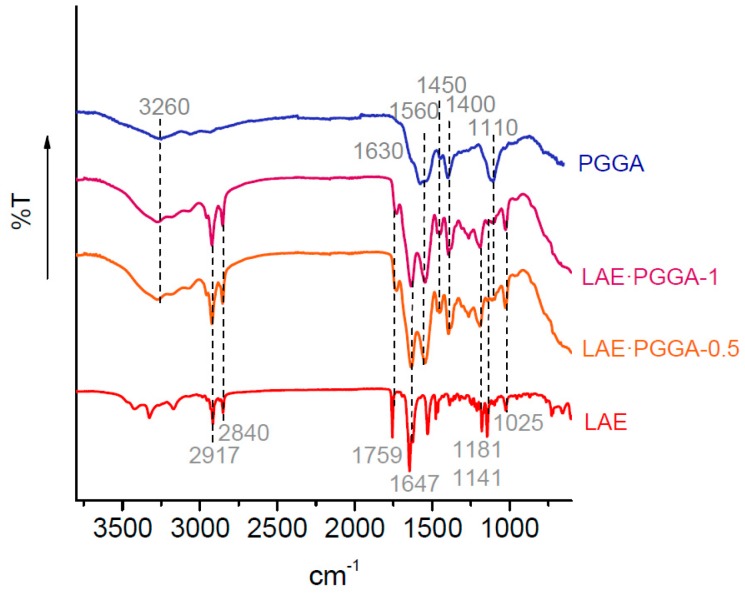
Compared FTIR spectra of LAE, PGGA, and LAE·PGGA complexes.

The presence of the two components in the LAE·PGGA complexes was evidenced by FTIR, as it is shown in Figure 1. The characteristic absorptions of both LAE and PGGA are present in the spectra of the complexes with the expected relative transmittance values.

**Figure 2:**
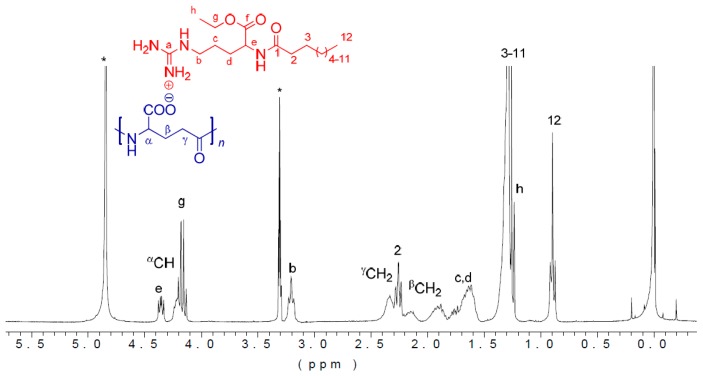
^1^H NMR spectra of LAE·PGGA-1 recorded at 25 °C in MeOD. *Asterisked signals are those arising from water and non-deuterated solvent.

The ^1^H NMR analysis of the complexes ascertained the presence of the two components and provided an accurate quantification of their composition. The spectrum recorded from LAE·PGGA-1 is depicted in Figure 2, with indication of peak assignments. The area ratio of the signal arising from the γ-methylene of PGGA to the area of the two partially overlapped signals, including the 3-11 methylenes of the lauroyl chain of LAE revealed that the actual composition of the complexes LAE·PGGA-1 and LAE·PGGA-0.5 were 0.9:1 and 0.5:1, respectively, which are values that are very close to those expected from the relative amounts of the two components that were used for their preparation. The ^1^H NMR spectrum of LAE·PGGA-0.5, as well as the ^13^C NMR of the two complexes are shown in the Supporting Information (SI) file associate to this paper (Figures SI-1 and SI-2 in the supplementary materails).

### 3.3. Thermal Properties and Structure

**Figure 3:**
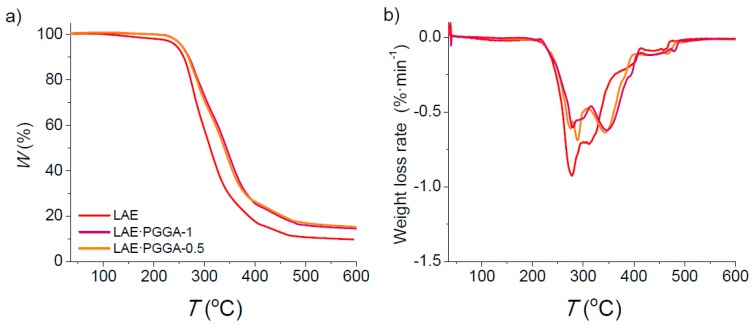
Thermogravimetric analysis (TGA) traces (**a**) and derivative curves (**b**) of LAE and its ionic complexes with PGGA.

The thermal decomposition of the LAE·PGGA complexes was examined by TGA under inert atmosphere, and the possible thermal transitions were explored by DSC. The TGA traces recorded for the two complexes as well as their respective derivative curves are compared with that of LAE in Figure 3, and decomposition temperatures and remaining weights measured on these traces are given in Table 2.

It was found that decomposition of the complexes started about 10 °C above that of LAE and that the whole process happened through two stages in the three cases. Temperatures at which the first decomposition step took place at maximum rate were not very dissimilar for the three samples, whereas values that were observed for the second step were much higher for the complexes than for LAE. The thermal decomposition of ionic complexes of PGGA with trimethylalkylammonium surfactants (*n*ATMA·PGGA) has been studied by us in some detail [37]. It was then reported that *n*ATMA·PGGA started to decompose around 200 °C by the decoupling of the complex, and that decomposition proceeded along two steps at rate temperatures within the 270–280 °C and 320–380 °C ranges, respectively. Given the resemblance of the overall thermal decomposition patterns of LAE·PGGA and *n*ATMA·PGGA, a similar mechanism may be assumed to occur in both types of complexes, in spite that decomposition of LAE should be expected to be much more intricate than that of alkylammonium compounds.

### 3.4. Supramolecular Structure and Thermal Transitions

**Figure 4:**
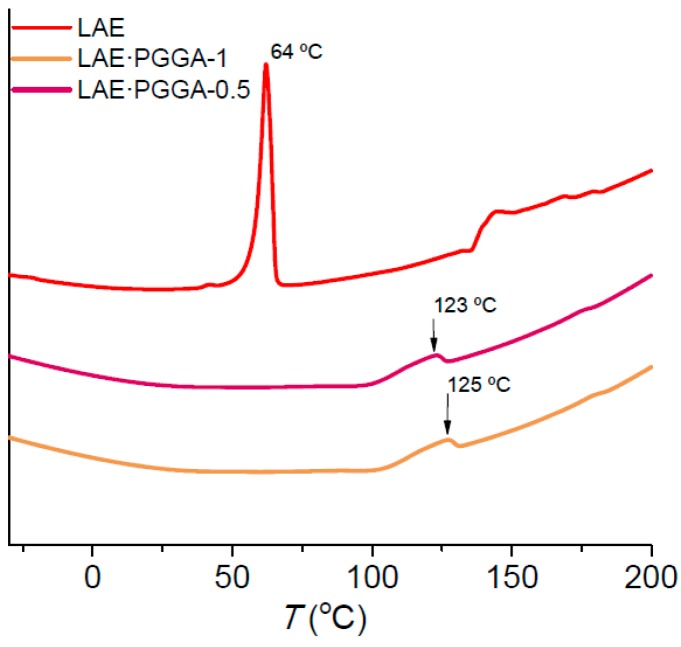
Differential scanning calorimetry (DSC) traces of LAE and its ionic complexes with PGGA.

**Figure 5:**
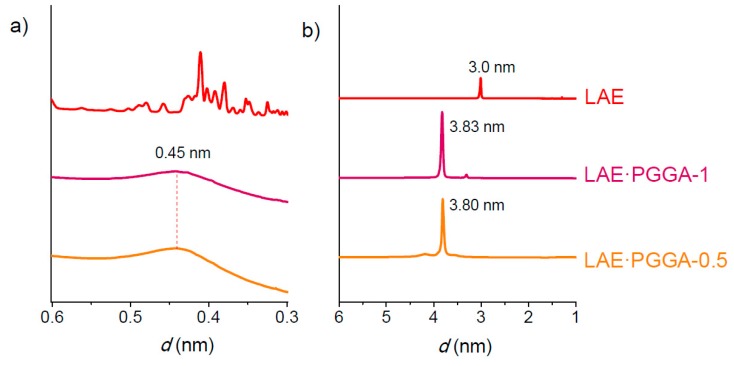
XRD profiles recorded from LAE and its complexes with PGGA in the wide-angle region (WAXS) (**a**) and small angle regions (SAXS) (**b**) at room temperature.

The DSC traces recorded at heating from LAE and the complexes in the 0–200 °C range are depicted in Figure 4. A well-defined sharp endothermal peak was observed for LAE at 62 °C, which doubtlessly arises from melting. In fact, the XRD of LAE in the WAXS produced multiple discrete scattering in the 0.3–0.5 nm range characteristic of crystalline organic material (Figure 5a). Furthermore, the examination of this sample under the polarizing optical microscope (POM), while heating revealed the presence of a typical crystalline texture that disappeared at temperatures nearly above 60 °C (see Figure SI-3 in the SI file in the supplementary materails). On the contrary, the DSC traces of both LAE·PGGA-1 and LAE·PGGA-0.5 did not show below 100 °C any sign that was indicative of crystallinity. Accordingly, no definite diffraction peak, but a broad peak centered on 0.45 nm characteristic of disordered material, was the only scattering detected in the WAXS of the complexes, which is taken as indicative that the LAE counterpart must be in the amorphous state.

Inspection of the SAXS of the XRD patterns revealed in every case the presence of one sharp diffraction peak corresponding to a spacing of 3.0 nm for LAE and of ~3.8 nm for the complexes (Figure 5b). The presence of a peak in the ~3–4 nm range of SAXS is a distinctive characteristic of the ionic complexes made of PGGA and tetraalkylammonium surfactants bearing long linear alkyl chains that are arranged in a biphasic structure made of alternating polypeptidic and paraffinic layers [12,13]. According to such antecedents, the ~3.8 nm spacing observed for both LAE·PGGA-1 and LAE·PGGA-0.5 can be interpreted as arising from the periodicity of the biphasic layered arrangement that was adopted by these complexes, although the LAE moiety is in the non-crystallized state. The long spacing displayed by LAE·PGGA (~3.8 nm) is consistent with that observed for LAE (3.0 nm), since the space occupied by the PGGA layer has to be added in the complex. It is also in agreement with the long spacing reported for 12ATMA·PGGA (3.1 nm) [13] provided that the LAE non-alkyl moiety is much bulkier than the trimethylammonium group of 12ATMA.

**Figure 6:**
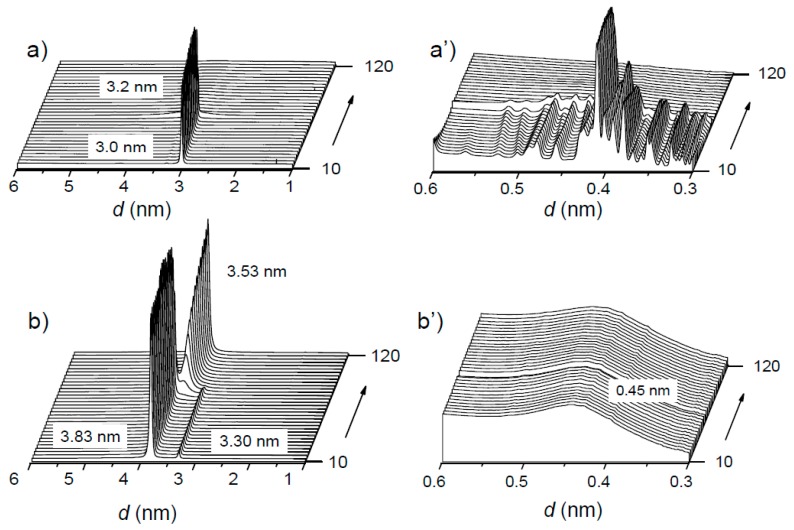
Evolution of the SAXS and WAXS profiles of LAE (**a**,**a’**) and LAE·PGGA-1 (**b**,**b’**) at heating from 10 to 120 °C.

In order to have a deeper insight into the structure of the LAE·PGGA complexes, an XRD study at variable temperature was carried out using synchrotron radiation and the spacing data measured in this study are listed in Table 3. Both WAXS and SAXS traces were simultaneously registered at real time from samples while heated or cooled over the 10–120 °C range at a rate of 10 °C·min^−1^. The evolution of the SAXS and WAXS profiles recorded for LAE is shown in Figure 6a,a’. The scattering that was initially present in both regions was retained until heating up to 60 °C to completely disappear at higher temperatures in full agreement with what was observed by DSC. No changes were detected after cooling (Figure SI-4, SI file in the Supplementary Materials), confirming the incapacity of LAE to crystallize from the melt, such as was evidenced before by both DSC and POM.

The results obtained in the thermal XRD study of LAE·PGGA-1 are shown in Figure 6b,b’. In this case, the SAXS peak at 3.83 nm was kept almost invariable over the whole temperature interval, indicating that the layered arrangement present in the complex was essentially retained at the applied temperatures. In the WAXS region, the broad peak that was observed at 0.45 nm was unaffected by heating as it should be expected for a disordered scattering. It should be noted, however, that a small jump of the 3.83 peak down to 3.53 nm was observed around 60 °C. The occurrence of small jumping in the SAXS peaks in the 30–60 °C range is a frequently observed fact in the heating of comb-like ionic complexes of PGGA. Jumping may be either upwards or downwards, and it is invariably attributed to the occurrence of small rearrangements that take place in the layered structure upon melting of the alkyl chain [38].

The jump observed here for LAE·PGGA-1 cannot be explained in the same manner as for *n*ATMA·PGGA complexes, since the alkyl chain of LAE is not crystallized. It may be speculated however that some spatial rearrangement could occur in the molecular assembly of the LAE nanophase involving a light shortening of the interlayer distance of the complex. In this regard, it is interesting to notice that no jump was observed for LAE·PGA-0.5 (Figure SI-5 in SI file in the supplementary materials) where the low concentration of LAE may be insufficient to adopt a close continuous packing in this phase. POM observations of LAE·PGGA-1 subjected to heating revealed an initial typical liquid-crystalline texture at room temperature that slightly changed above 60 °C to fully disappear when the temperature reached the proximities of 125–130 °C (Figure SI-6 in SI file in the supplementary materials). This behavior is in agreement with DSC results that showed the presence of a small peak at 123 °C characteristic of a liquid crystal-isotropic phase transition.

### 3.5. LAE Release and Antibacterial Properties

**Figure 7:**
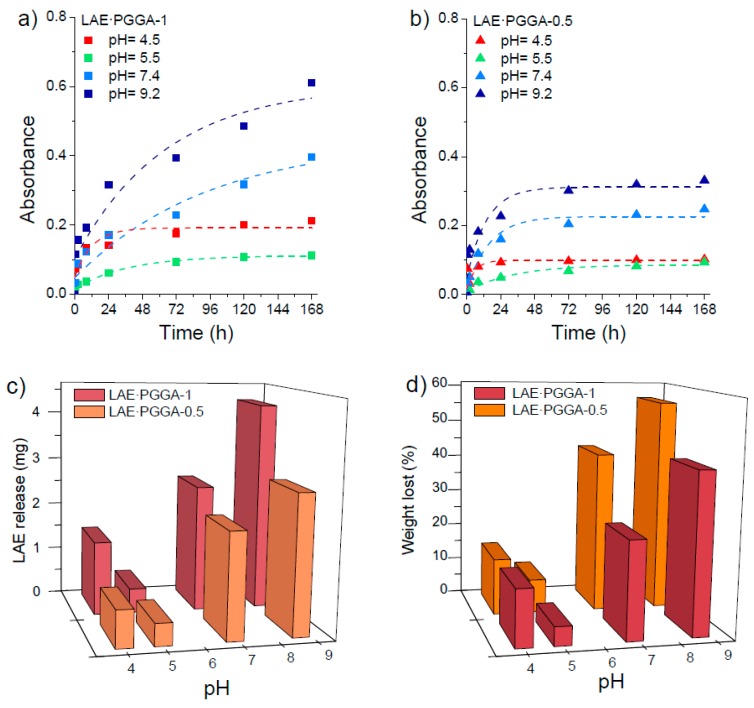
Dissociation of LAE·PGGA-1 (**a**) and LAE·PGGA-0.5 (**b**) complexes in aqueous buffer at the indicated pH. In (**c**,**d**) the amount of released LAE and the film weight loss are respectively compared for the two complexes after seven days of incubation at the assayed pH.

Although it has been reported that PGGA is a moderate microbiocide [16], it is the LAE counterpart of the LAE·PGGA complexes that is expected to play the main biocide activity in these systems. Accordingly, the biocide activity of the LAE·PGGA films in aqueous medium will be largely determined by the LAE concentration that is attained in the environment upon dissociation of the complex. To substantiate this hypothesis the accumulative amount of LAE that is released from the LAE·PGGA films to the incubation medium at 25 °C was estimated by measuring the absorbance at 220 nm of the supernatant solution as a function of time. The results obtained from these assays for both LAE·PGGA-1 and LAE·PGGA-0.5 at different pH ranging from ~4.5 to ~9.5 are shown in Figure 7a,b As it could be logically expected, the general trend is that the amount of LAE present in the buffer increased exponentially with time to finally reach a more or less constant concentration. The influence of pH on the delivery of LAE is clearly illustrated in the bar graphics shown in Figure 7c,d. In these plots, both the amount of LAE that is present in the incubation medium and the weight lost by the film is compared for the two complexes after one week of incubation at the different assayed pH. It is clearly seen that LAE is liberated much faster at basic pH and that the minimum release rate happens at pH 5.5, a result that may be explained by taking into account the pKa of the two complex components, i.e., PGGA and LAE. It is also evidenced that the liberated amount of LAE is higher in LAE·PGGA-1 than in LAE·PGGA-0.5, which is much according to expectations, whereas the weight that is lost by the latter is significantly greater, a difference that is more ostensible at pH 9.2. This apparent conflict may be rationalized by taken into account the partial hydrolysis that it is probable undergone by PGGA upon incubation. The hydrolytic degradation of PGGA is a well-known process that is favored at higher pH [39]. This process is expected to happen more extensively in the case of LAE·PGGA-0.5 due to the higher accessibility of the PGGA backbone to water in this complex.

**Figure 8:**
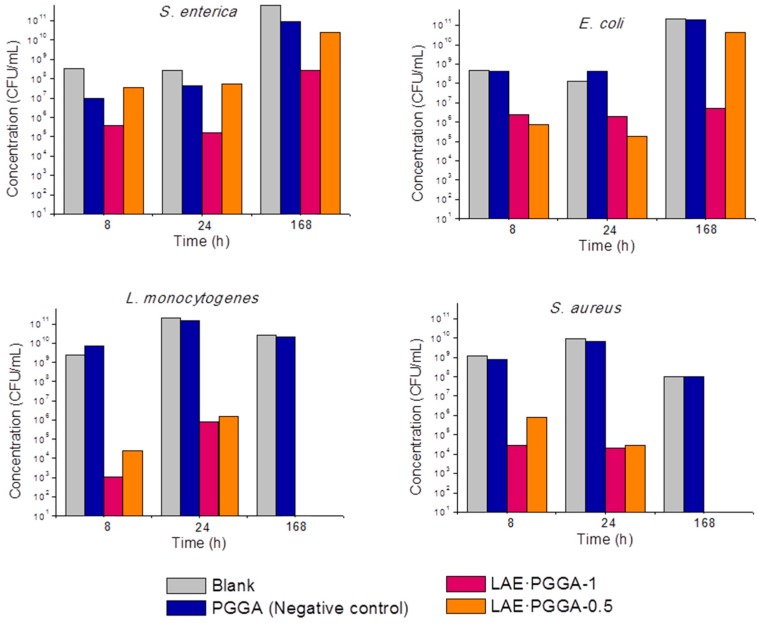
Antibacterial activity of LAE·PGGA-1 and LAE·PGGA-0.5 films against Gram-negative and Gram-positive bacteria expressed as concentration of colony forming units (CFU·mL^−1^).

The antibacterial activity of LAE·PGGA complexes against both Gram-negative (*S. enterica* and *E. coli*) and Gram-positive bacteria (*L. monocytogenes* and *S. aureus*) as a function of time was evaluated by the liquid medium method. Single colony of each strain was suspended into the buffer placed in essay tubes, and the incubation effects were followed both visually and by measuring the optical density. The turbidity appreciated by visual appearance of the supernatant was a preliminary indication of how the bacterial growing is affected by the presence of the complex (Figure SI-7 in the SI file in the supplementary materials). The results that were obtained by spectroscopic measurement are graphically depicted in Figure 8, together with those obtained for neat PGGA (negative control NC), and the blank. Average values that were obtained from triplicate counting and calculations are presented. Numerical data of these results expressed as Log(CFU) (logarithm of colony forming units), as well as their corresponding logarithmic reduction values (LRV) and reduction percentages (PR) calculated by means of the expressions given above are given in Table 4.

An overall inspection of the results obtained in the biocide activity assays leads us to conclude that, in agreement with the amount of LAE that is delivered in each case, the bactericide effect is in general more pronounced for LAE·PGGA-1 than for LAE·PGGA-0.5. The higher bacterial growth reduction capacity observed for the complex containing more LAE is according to previous observations that were made on antimicrobial films based on LAE loaded chitosan [27].

The two complexes displayed a similar great biocide activity against Gram-positive bacteria, reaching an almost total growth inhibition for both *L. monocytogenes* and *S. aureus* after seven days of incubation, and >99.99% of reduction after 24 h. On the contrary, both of the complexes were less effective against Gram-negative bacteria *(S. enterica* and *E. coli*), which is agreement with observations made by other authors on the antibacterial activity of LAE [27,40]. The LAE·PGGA-0.5 films achieved only 2.3% reduction of *S. enterica* after 24 h and 70.9% after seven days, although the double concentrated complex reached 99.6% reduction of the same bacteria after 24 h. Since Gram-negative organisms have a greater defense system due to the outer lipopolysaccharide coat that surrounds the cell wall, the diffusion of hydrophobic compounds is expected to be much more hindered than in Gram-positive species [41].

Since our results were obtained from one single experiment and only one strain was used for each bacterium, they should be taken as a first evaluation of the bactericide potential of these complexes. Further studies, including different incubation conditions (pH, temperature, etc.), additional microorganisms, and a statistical analysis of data collected from replicated experiments, will be required to attain a more definite appraisal of the biocide capacity of the LAE·PGGA system. Furthermore, the possibility that a VBNC (viable but non-culturable) state is adopted by the bacteria upon the action of the bactericide cannot be completely discarded since it has been described for some of the pathogens studied in this work [42]. In this case, however, such a situation is highly improbable since LAE is a biocide in use for long time, and, to our knowledge, the occurrence of dormant bacteria after the action of this agent has not been reported.

## 4. Conclusions

The widely known antimicrobial agent LAE (ethyl ^α^*N*-lauroyl l-arginate) was ionically coupled with poly(γ-glutamic acid) (PGGA) to generate host-guest ionic complexes containing either stoichiometric molar amounts of the two components or an half amount of LAE. The complexes may be used to prepare consistent films that are non-water soluble and are thermally stable. Both complexes adopt a layered biphasic structure similar to that described for similar ionic complexes of PGGA made of alkyltrimethylammonium surfactants. Although LAE is a crystalline compound, the LAE·PGGA complexes are essentially amorphous with the lauroyl chains staying in a disordered state. LAE is released from the complexes upon incubation in aqueous buffer at a rate that is depending on pH and in less degree on the LAE/PGGA ratio. Films of these complexes displayed antibacterial activity against both Gram positive and Gram-negative strains, although the biocide effect on the former was much more ostensible. The biocide effect is motivated by both the LAE released to the aqueous environment and the fraction bounded to the complex. Since the two components that integrate the complexes are edible, these complexes offer interesting potential as antibacterial materials to be used in both food additives or packaging.

## Figures and Tables

**Table 1:** Results for the preparation of LAE·PGGA complexes.

Complex	LAE:PGGA ^a^	Mixing Conditions		Yield (%)	Color	Composition ^d^
		*c* (M) ^b^	*T* (°C) ^c^			
LAE·PGGA-1	1.0:1.0	0.01	35	70	white	0.9:1.0
LAE·PGGA-0.5	0.5:1.0	0.01	35	57	white	0.5:1.0

^a^ Molar ratio of LAE to PGGA used for coupling; ^b^ Concentration of the solutions mixed to form the complex; ^c^ Minimum temperature at which LAE is soluble in water at the used concentration; ^d^ Molar ratio of LAE to PGGA in the complex determined by ^1^H NMR.

**Table 2:** Thermal parameters of LAE·PGGA complexes.

Sample	°*T*_d_^a^ (°C)	^max^*T*_d_^a^ (°C)	*W* ^a^ (%)	*T*_m_ (°C)
LAE	245	275/311	27/10	62
LAE·PGGA-1	255	280/349	68/15	-
LAE·PGGA-0.5	253	273/342	63/16	-

^a^ °*T*_d_ and ^max^*T*_d_ are onset and maximum rate decomposition temperatures, respectively; *W* is the remaining weight at the end of each decomposition process; ^b^ *T*_m_ is the melting temperature recorded by DSC.

**Table 3:** X-ray diffraction data of LAE·PGGA complexes.

Sample	SAXS			WAXS		
	*L*_0_^10 °C^	*L*_0_^120 °C^	*L*_0_^10 °C^	*d*^10 °C^	*d*^120 °C^	*d*^10 °C^
LAE	3.0	-	-	Multiple	-	-
LAE·PGGA-1	3.8	3.5	3.5	0.45	0.45	0.45
LAE·PGGA-0.5	3.8	3.5	3.5	0.45	0.45	0.45

*L*_0_: interlamellar distance (layered structure window); *d*: interplanar distances arising from Bragg spacings

**Table 4:** Bactericide activity of films made of LAE·PGGA complexes against Gram-negative and Gram-positive bacteria after 1 and 7 days of incubation at pH 7.4 and 37 °C.

		*S. enterica*		*E. coli*		*L. monocytogenes*		*S. aureus*	
		24 h	7 d	24 h	7 d	24 h	7 d	24 h	7 d
PGGA	Log(CFU) ^a^	7.6	10.6	8.6	11.3	11.2	10.30	9.8	8.0
LAE·PGGA-1	Log(CFU)	5.2	8.4	6.3	6.7	3.9	total	4.3	0.3
	LRV ^b^	2.4	2.5	2.3	4.6	7.3		5.5	7.7
	PR (%) ^c^	99.6	99.7	99.5	>99.9	>99.9	100	>99.9	>99.9
LAE·PGGA-0.5	Log(CFU)	7.6	10.4	5.3	10.6	4.2	total	4.5	total
	LRV	0.01	0.5	3.3	0.6	7.0		5.4	
	PR (%)	2.3	70.9	99.9	76.8	>99.9	100	>99.9	100

^a^ Antibacterial activity expressed as logarithm of colony forming units; ^b^ Antibacterial activity expressed as log reduction value; ^c^ Percentage of reduction.

## Supplementary Materials

The following are available online at www.mdpi.com/2073-4360/10/1/21/s1.
