# Supplementary material for: Antibacterial Films Made of Ionic Complexes of Poly(γ-glutamic acid) and Ethyl Lauroyl Arginate
Source: Polymers (Basel). 2017 Dec 24;10(1):21. doi: 10.3390/polym10010021 (PMC6414889; doi:10.3390/polym10010021)
Supplement: Supplementary file 1 [file polymers-10-00021-s001.docx]

**Supplementary Materials:**

**Antibacterial Films made of Ionic Complexes of Poly(γ-glutamic acid) and Ethyl Lauroyl Arginate**

Ana Gamarra^a^, Beatriz Missagia^b^, Jordi Morató^b^, Sebastián Muñoz-Guerra^a^*

^a^Departament d’Enginyeria Química, Universitat Politècnica de Catalunya,

ETSEIB, Diagonal 647, Barcelona 08028, Spain

^b^Health and Environmental Microbiology Lab & UNESCO Chair on Sustainability, Universitat Politècnica de Catalunya, ESEIAAT, Ediﬁci Gaia, Pg. Ernest Lluch/Rambla Sant Nebridi, Terrassa 08222, Spain

E-mails: [anagamarramontes@gmail.com](mailto:anagamarramontes@gmail.com); [beatrizmissagia@gmail.com](mailto:beatrizmissagia@gmail.com); [jordi.morato@upc.edu](mailto:jordi.morato@upc.edu)

Corresponding author: E-mail: [sebastian.munoz@upc.edu](mailto:sebastian.munoz@upc.edu); Tel: 34 934016680

**Table of contents**

**Figure S1.** ^1^H NMR spectra of LAE·PGGA-0.5 recorded at 25 ºC in MeOD. *Asterisked signals are those arising from water and non-deuterated solvent.

Figure S2. ^13^C NMR spectra of LAE·PGGA-1 (a) and LAE·PGGA-0.5 (b) recorded at 25 ºC in MeOD.

Figure S3. POM micrographs recorded from LAE at heating (a,b,c) and after heating (d) at the indicated temperatures.

**Figure S4.** Evolution of the SAXS and WAXS profiles of LAE (a and a´) and LAE·PGGA-1 (b and b´) at cooling from 120 to 10 ºC.

**Figure S5.** SAXS (a) and WAXS (b) profiles of LAE·PGGA-0.5 at heating (left) and cooling (right).

Figure S6. POM micrographs recorded from LAE·PGGA-1 at heating (a,b,c) and after heating (d) at the indicated temperatures.

**Figure S7.** Visual appearance of the supernatant of LAE·PGGA-1 and LAE·PGGA-0.5 films incubated with *Listeria monocytogenes* (LM), *Staphylococcus aureus* (SA), *Salmonella enterica* (SE) and *Escherichia coli* (EC).

**Figure S1.** ^1^H NMR spectra of LAE·PGGA-0.5 recorded at 25 ºC in MeOD. *Asterisked signals are those arising from water and non-deuterated solvent.

Figure S2. ^13^C NMR spectra of LAE·PGGA-1 (a) and LAE·PGGA-0.5 (b) recorded at 25 ºC in MeOD.


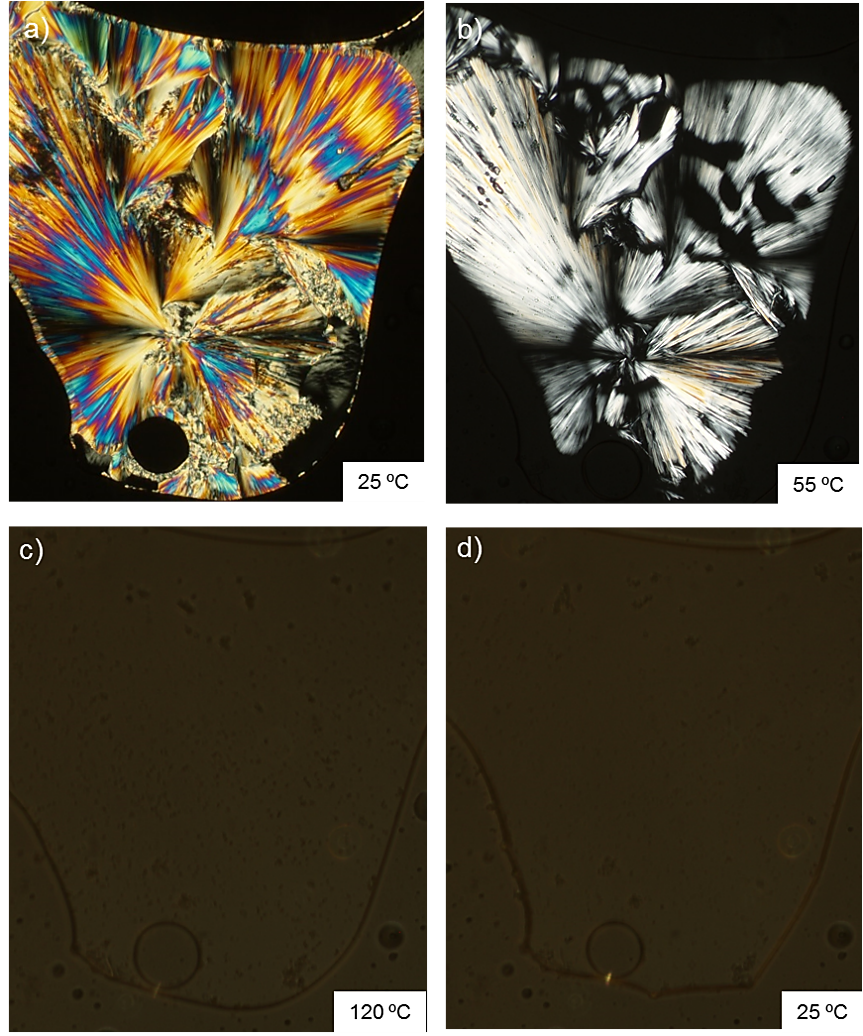


Figure S3. POM micrographs recorded from LAE at heating (a,b,c) and after heating (d) at the indicated temperatures.

**Figure S4.** Evolution of the SAXS and WAXS profiles of LAE (a and a´) and LAE·PGGA-1 (b and b´) at cooling from 120 to 10 ºC.

**Figure S5.** SAXS (a) and WAXS (b) profiles of LAE·PGGA-0.5 at heating (left) and cooling (right).


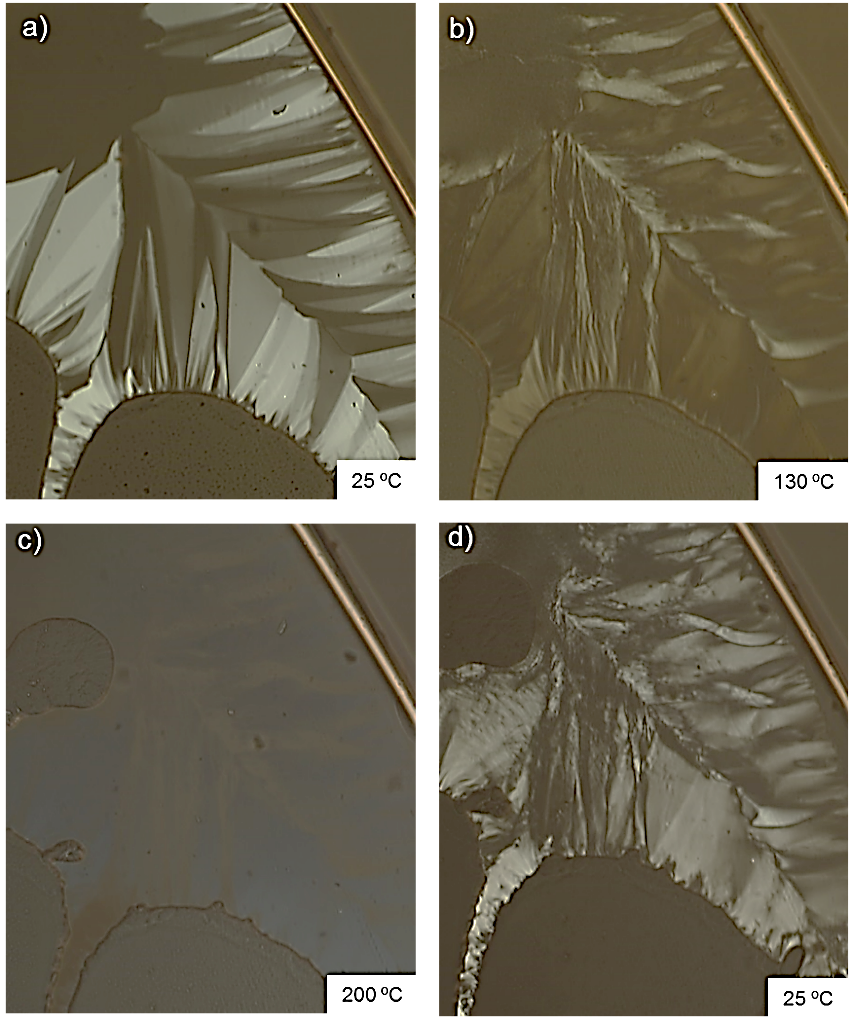


Figure S6. POM micrographs recorded from LAE·PGGA-1 at heating (a,b,c) and after heating (d) at the indicated temperatures.


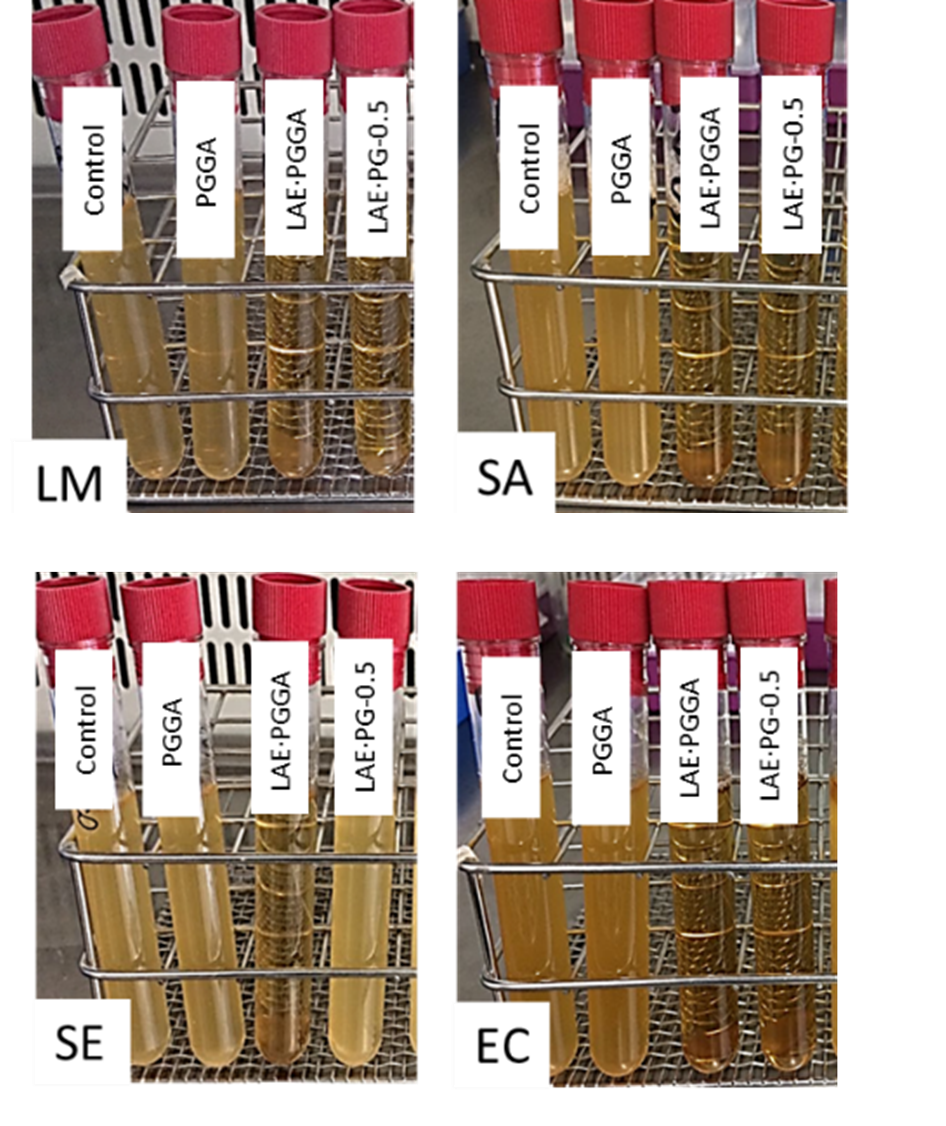


**Figure S7.** Visual appearance of the supernatant of LAE·PGGA-1 and LAE·PGGA-0.5 films incubated with *Listeria monocytogenes* (LM), *Staphylococcus aureus* (SA), *Salmonella enterica* (SE) and *Escherichia coli* (EC).
